# The prevalence of the culturable human skin aerobic bacteria in Riyadh, Saudi Arabia

**DOI:** 10.1186/s12866-019-1569-5

**Published:** 2019-08-16

**Authors:** Ashwag Shami, Samiah Al-Mijalli, Pisut Pongchaikul, Ahmed Al-Barrag, Samah AbduRahim

**Affiliations:** 1 Biology Department, College of Sciences, Princess Nourah bint Abdulrahman University, Riyadh, Saudi Arabia; 2 Chakri Naruebodindra Medical Institute, Faculty of Medicine Ramathibodi hospital, Mahidol University, Bangkok, Thailand; 3 Department of Pathology, Medical Microbiology, College of Medicine, King Saud University, Riyadh, Saudi Arabia; 4 Department of Microbiology, Faculty of Medical Laboratory Sciences, University of Khartoum, Khartoum, Sudan

**Keywords:** Microbiota, Skin, Aerobic bacteria, Staphylococci, Diversity

## Abstract

**Background:**

Human skin is an appropriate environment for the growth of different types of microbes that may inhabit the skin as commensal flora. This study aims at identifying the diversity of skin microbiota in healthy Saudi population. In this study, 80 Saudi subjects of both males and females, from different habitat, and different ages (elderly and young), were recruited to determine the aerobic bacterial flora from their three skin sites; hand, scalp and foot. A single colony obtained from aerobic culture was identified using Biomérieux VITEK® 2 system. For those not being identified by VITEK® 2 system, the identification was conducted using 16 s rRNA sequence.

**Results:**

Thirty-three bacterial species were isolated from males, whilst 24 species were isolated from females. Micrococci are the predominant organisms, followed by Staphylococci, Pantoea species, and lastly *Enterococcus faecium*. *Acinetobacter baumannii, Enterococcus faecalis,* and *Klebsiella pneumoniae* were only found in elder subjects, while *Pseudomonas aeruginosa* was isolated from the young only. The number of bacterial isolates in the elders was higher that of the young. The average number of flora was larger in foot, then hand and lastly scalp.

**Conclusion:**

Here we show the difference in the number of cultivable bacteria across age and gender that may result in the variety of local skin infection. This study paves the way to further investigation in the aspect of in-depth metagenomics analysis and host-pathogen interaction.

**Electronic supplementary material:**

The online version of this article (10.1186/s12866-019-1569-5) contains supplementary material, which is available to authorized users.

## Background

Skin microbiota introduces the entire pool of microbes, which comprise bacteria, archaebacteria, fungi, virus and mites [1]. The variation of skin microbiota is seen in different parts of the body and between individuals [2]. Microbes that colonise skin could be influenced by skin microenvironment, such as moisture, sebaceous environment, by intrinsic features such as the age and gender, and by extrinsic features such as clothing, hygiene, humidity and occupation [3].

Previously, the microbes found on skin are often considered pathogenic organisms or symbiotic organisms. Nevertheless, recent data suggested that skin microbes, in healthy skin, play a role in host defence [4]. The chemical and physical factors of the skin could be the main reason for the adaptation of unique groups of microorganisms to inhabit specific region of the skin [5]. The arrangement of the skin surface differs due to regional variety in skin anatomy; culture-based studies indicated that these regions are identified to support distinct groups of microorganisms [2]. Features specific to the host, such as age, sex and location, also affected the diversity of the bacteria on the skin. Age has a great influence on the microenvironment of the skin and, consequently, on the colonising microbiota. The commence of microbiota colonisation in human remains under the argument. The colonisation can occurs directly after birth, either through vaginal delivery or in the minutes after birth by caesarean section, or it happens in utero [6, 7]. Previous studies demonstrated that healthy microbiota of the skin varies depending on microenvironment of the skin. Moreover, other individual factors, such as age and gender, have an impact on skin microbiota [8]. In Saudi Arabia there are very limited researches about the human microbiota. We have compiled three studies, one of which was on microbiota collected from the reproductive tracts of women [9], and the other two on gut microbiota [10, 11]. but, according to our knowledge this is the first work regarding the aerobic skin flora of Saudis. One of the main goals of this study is to identify bacterial fingerprint for Saudi community and to study the relation of these flora microbiota to diseases.

## Results

**Fig. 1:**
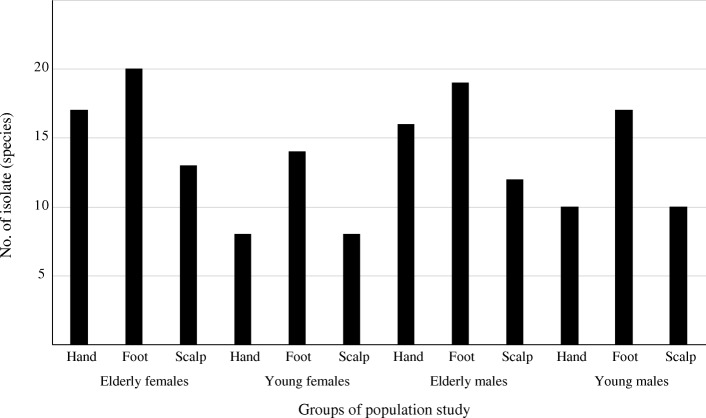
A bar chart illustrates the numbers of cultivable bacteria isolated from hands, hair and feet of elder female, young female, elder male, and young male

Eighty Saudi subjects from both sex groups (males and females), different habitat, and different ages (elder and young), were screened to determine the aerobic bacterial flora from their three skin sites; hand, scalp and foot (Additional file 1: Table S1). Biochemical tests were performed to isolate the bacteria along with molecular techniques. The results of all isolates that were identified using 16 s rRNA sequencing were in Genus *Micrococcus.* Thirty-three bacterial species were isolated from males, whilst 24 species were isolated from females. The sites and the total numbers of bacteria isolated from the different sites of subjects are illustrated in Fig. 1. The majority of bacteria were isolated from foot, followed by the hand and scalp. A one – factor ANOVA showed the significant difference of the amount of culturable bacteria from three different sites (scalp, hand and foot) (*p* < 0.05).

There was a significant effect of age and gender on the number of bacteria isolated for the four groups. By conducting post – hoc analysis, the mean numbers of culturable bacteria isolated from elder subjects (2.39 ± 0.99) was higher than young’s (1.82 ± 1.12) at the *p* < 0.05 level. The higher number of culturable isolates was found in old females (2.6 ± 0.94), in comparison with young females (1.45 ± 1.05). However, pairwise comparison of other pairs did not reveal significant difference. Taken together, these results suggested that the number of culturable aerobic bacteria varied by site, age and gender. Specifically, the foot of old females contained the highest number of culturable aerobic bacteria.

Out of 43 species identified, 19 species were isolated from male only and 10 species were from female only. The organisms isolated from both were listed in Table 1. *Micrococcus spp.* are the predominant organisms, followed by *Staphylococcus spp.*, and *Pantoea spp*. *Acinetobacter baumannii, Enterococcus faecalis,* and *Klebsiella pneumoniae* were exclusively isolated from elder subjects, while *Pseudomonas aeruginosa* was isolated from the young only. The number of isolates obtained from the elderly were more than that of the young. Bacteria isolated only from elderly males represented 56% of total bacteria isolated from males (Additional file 1: Table S3) and bacteria isolated from elderly females represented 62.5% of total bacteria isolated from females (Additional file 1: Table S4).

**Table 1:** A total number of bacteria commonly isolated from study population

Isolate species	Elderly females	Young females	Elderly males	Young males
*Acinetobacter baumannii*	1	–	2	–
*Enterococcus faecalis*	1	–	1	–
*Enterococcus faecium*	–	1	1	–
*Klebsiella pneumoniae*	1	–	1	–
*Kocuria kristinae*	4	1	2	–
*Micrococcus spp.*	9	12	11	15
*Pantoea spp*	3	2	5	–
*Pseudomonas aeruginosa*	–	1	–	2
*Pseudomonas luteola*	1	–	2	2
*Sphingomonas paucimobilis*	1	–	2	2
*Staphylococcus epidermidis*	4	4	1	1
*Staphylococcus haemolyticus*	11	–	–	2
*Staphylococcus hominis*	2	2	1	1
*Staphylococcus warneri*	3	1	1	–
Total	41	24	30	25

## Discussion

The aerobic skin microbiota from three different sites of skin; scalp, hand and foot were investigated in 80 Saudi subjects live in Riyadh in a diversification of age and gender. There is a marked variation at the level of species, as some species were isolated exclusively from certain site and not isolated from others, however, other species have been isolated from two or even the three sites. The average number of flora was larger in foot, then hand and lastly scalp. There was also no significant difference between male and female subjects in the average number of overall detected bacteria. This finding was contrary to previous studies done by Ying et al. and Haro et al. which proved the differences in the bacterial community structure significantly related to gender [12, 13]. Interestingly, the mean numbers isolated only from elder subjects was significantly higher (*p* < 0.05) than young’s, (Additional file 1: Tables S3 and S4). At this outcome, a preceding study carried out by Leyden et al. reinforces our findings that the quantitative levels of resident aerobic and anaerobic bacteria of the face show a characteristic age-related pattern [14]. Furthermore, studies reported that the age of the individual was found to influence the bacterial flora [15, 16]. It is important to note here that these previous findings apply only to ours, regarding elder women, but intriguingly, multiple comparison detect no significant difference between elder males, young males and young females.

Out of 43 diverse organisms, Micrococci and Staphylococci are the most frequently isolated organisms. Congruently**,** Somerville investigated the normal flora of the skin in different age groups. It was found that coagulase negative staphylococci and micrococci were found in, virtually, all skin sites in each person [15]. With few exceptions, the bacteria isolated in this work are well documented as human pathogens in healthy and immunocompromised individuals and some are reported as emergent pathogens. Interestingly, several well-known species, including *E. faecium, S. aureus, K. pneumoniae, A. baumannii, P. aeruginosa, and Enterobacter spp.*, were observed as skin flora in this study. These species might be environmental organisms or hospital-associated organisms. It is believed that close contact can provide the transmission of skin colonisers; however, there is no strong evidence indicating skin infection caused by skin flora.

By investigating Genus Enterobacter, *E. cloacae,* a well-known nosocomial pathogen, was identified from the skin culture [17]. Enterococci are opportunistic pathogens in individuals with serious diseases whose immune systems are compromised and in patients who have been hospitalised for prolonged periods or who have received broad-spectrum antimicrobial therapy [18]. There were three species of Enterococci identified: *E. faecalis*, *E. faecium*, and *E. casseliflavus*. *E. faecalis* and *E. faecium* were obtained from both males and females. Nevertheless, *E. casseliflavus* was found from hands and feet in male. Even though the species was reported associated with biliary tract disease in human, the infection caused by *E. casseliflavus* remained unknown [19, 20].

Coagulase-negative staphylococci (CoNS) becomes more important in nosocomial infections [21]. In exception of *S. lentus*, an animal pathogens which was recently reported to cause peritonitis in human [22], all CoNS were known to cause human infections*.* For example, *S. capitis* was reported to be associated with septic arthritis in prosthetic joint [23]. *S. gallinarum* was found associated with traumatic endophthalmitis after iron nail injury [24]. Interestingly, *S. saprophyticus*, which is a common skin commensal that is associated with urinary tract infection in women, was absent in the present study [25].

For unusual species, *Escherichia hermannii* and *Klebsiella oxytoca* were isolated from male elderly subjects only. The former was mainly found in environment. However, it was also recovered from clinical samples (i.e. purulent wound and discharge) and it was found as both mixed and sole infectious isolates. The latter is considered an opportunistic pathogen that carries drug-resistance genes [26, 27].

According to our findings, there are species most likely to be found in less frequency in the healthy subjects. Notwithstanding, it is possible that low abundance skin microbes (< 1%) can be recovered by the culture [28]. Hence, further study using molecular investigation, such as metagenomic approach, should be applied to determine broader, especially, uncultivable species.

## Conclusion

Our results suggested that, bacterial diversity of flora is different across age and gender. The number of flora seen in elderly is more than that of the young. The foot contains the highest number of flora, followed by the hand and the scalp, respectively. Indeed, this study provides a preliminary step for researches on the skin microbiota of Saudis, however its findings do not reflect the final data. Therefore, the metagenomic analyses of skin flora, especially anaerobes, higher sample size and more cofactors variables will be considered in the future. The finding will pave the way for the study of association between flora and systemic diseases.

## Methods

### Study population

Eighty Saudi adults from Riyadh were enrolled in this study (Additional file 1: Table S1). They were divided into four groups according to their gender and age; elder females, elder males, young females and young males whose average ages were 62, 51, 20 and 21, respectively.

### Study area and sample collection

The study was conducted in the Princess Nourah bint Abdulrahman University and King Saud University Research Center in Riyadh. The samples were collected by sterile cotton swabs from most susceptible skin areas that contact with exogenous environmental factors and more susceptible pollution such as (hand, foot, and scalp), then were kept in 4 °C for less than 2 h. The sample collection process in this study was conducted for research purpose only.

### Identification of isolates

Swaps were inoculated on blood agar, tryptic Soy agar and MacConkey agar and incubated aerobically overnight at 37 °C. The bacteria were identified biochemically using Biomérieux VITEK® 2 system.

### Molecular identification

Bacteria that weren’t recognized by Biomérieux VITEK® 2 system were identified by 16 s rRNA. DNA was extracted and purified using a QIAGEN DNeasy Blood & Tissue kit according to the manufacturer’s instructions. 16 s rRNA universal primers (Additional file 1: Table S2) were used and PCR products were purified using ExoSAP-IT (Usb. Affymetrix, Inc). The PCR products were treated with ExoSAP-IT, and then subjected to 16 s rRNA sequencing (MOLECULE-ON, New Zealand).

### Data analysis

The data were analysed using R software. Descriptive statistics were performed for quantitative variables; the results were expressed as mean ± standard deviation in each group. One-Sample Kolmogorov-Smirnov and Levene statistic were carried out to test the distribution normality and Homogeneity of bacteria’s numbers respectively. We used one-way ANOVA to test the difference between the four groups (elder males, elder females, young males and young females). Because the variances of bacteria’s numbers among the three skin sites were unequal, we adopted Welch’s ANOVA, followed by Tukey test (to compare the means between groups). The mean difference is significant at the 0.05 level.

## Additional file

Additional file 1: **Table S1.** The summary of demographic data of healthy participants. **Table S2.** The DNA sequence of PCR primers for 16 s rDNA sequence in this study. **Table S3.** Species and numbers of bacterial flora isolated from males. **Table S4.** Species and numbers of bacteria isolated from females. (DOCX 28 kb)

## Data Availability

All data generated or analysed during this study are included in this published article and its Additional file.

## Abbreviations

ANOVA: Analysis of variance
CoNS: Coagulase-negative *Staphylococci*
PCR: Polymerase chain reaction
rRNA: Ribosomal RNA
