# Supplementary material for: The prevalence of the culturable human skin aerobic bacteria in Riyadh, Saudi Arabia
Source: BMC Microbiol. 2019 Aug 16;19:189. doi: 10.1186/s12866-019-1569-5 (PMC6697913; doi:10.1186/s12866-019-1569-5)
Supplement: Supplementary file 1 — Table S1. The summary of demographic data of healthy participants. Table S2. The DNA sequence of PCR primers for 16 s rDNA sequence in this study. Table S3. Species and numbers of bacterial flora isolated from males. Table S4. Species and numbers of bacteria isolated from females. (DOCX 28 kb) [file 12866_2019_1569_MOESM1_ESM.docx]

**Supplementary data**

**Table**

**Table S1: The summary of demographic data of healthy participants**

| Group | No. | Age (Mean ± SD, Median) | No. isolates (Mean ± SD) |
| --- | --- | --- | --- |
| Young female | 20 | 20.7 ± 2.13, 20 | 1.34 ± 1.10 |
| Elderly female | 20 | 62.3 ± 13.58, 61 | 2.6 ± 0.88 |
| Young male | 19 | 21.5 ± 3.85, 21 | 2.21 ± 1.17 |
| Elderly male | 21 | 51.04 ± 9.66, 49 | 2.19 ± 1.06 |

**Table S2: The DNA sequence of PCR primers for 16s rDNA sequence in this study**

| **Primer** | **Sequence** |
| --- | --- |
| 27F | 5’ AGAGTTTGATCMTGGCTCAG 3’ |
| 1492R | 5’ TACGGYTACCTTGTTACGACTT 3’ |

**Table S3: Species and numbers of bacterial flora isolated from males.**

| **Isolate species** | **Elderly males** | | | **Young males** | | |
| --- | --- | --- | --- | --- | --- | --- |
|  | **Hand** | **Foot** | **Scalp** | **Hand** | **Foot** | **Scalp** |
| *Acinetobacter baumannii* | - | 2 | - | - | - | - |
| *Enterobacter aerogenes* | - | - | - | - | 1 | - |
| *Enterobacter cloacae* | 1 | 1 | 1 | - | 1 | - |
| *Enterococcus casseliflavus* | 1 | 1 | - | - | - | - |
| *Enterococcus faecalis* | - | 1 | - | - | - | - |
| *Enterococcus faecium* | - | - | 1 | - | - | - |
| *Escherichia hermannii* | - | 1 | - | - | - | - |
| *Klebsiella oxytoca* | - | - | 1 | - | - | - |
| *Klebsiella pneumoniae* | 1 | - | - | - | - | - |
| *Kocuria kristinae* | 1 | - | 1 | - | - | - |
| *Kocuria rhizophila* | - | - | - | - | 1 | - |
| **Micrococcus spp* | 3 | 4 | 4 | 3 | 10 | 2 |
| **Micrococcus luteus* | - | - | - | 2 | 1 | 1 |
| *Pantoea spp* | 3 | 1 | 1 | - | - | - |
| *Pseudomonas aeruginosa* | - | - | - | - | 2 | - |
| *Pseudomonas luteola* | - | 1 | 1 | - | - | 2 |
| *Pseudomonas oryzihabitans* | - | 1 | - | - | - | - |
| *Pseudomonas putida* | - | - | - | - | 1 | - |
| *Pseudomonas stutzeri* | - | 1 | - | - | - | - |
| *Raoultella planticola* | - | - | 1 | - | - | - |
| *Rhizobium radiobacter* | - | 1 | - | - | - | - |
| *Serratia fonticola* | 1 | - | - | - | - | - |
| *Serratia odosifera* | 1 | - | - | - | - | - |
| *Sphingobacterium thalpophilum* | - | - | - | - | - | 1 |
| *Sphingomonas paucimobilis* | 1 | 1 | - | 2 | - | - |
| *Staphylococcus epidermidis* | - | - | 1 | 1 | - | - |
| *Staphylococcus gallinarum* | 1 | - | - | - | - | - |
| *Staphylococcus haemolyticus* | - | - | - | 1 | - | 1 |
| *Staphylococcus hominis* | 1 | - | - | - | - | 1 |
| *Staphylococcus lentus* | 1 | 1 | - | - | - | 1 |
| *Staphylococcus saprophyticus* | - | - | - | 1 | - | 1 |
| *Staphylococcus simulans* | - | 1 | - | - | - | - |
| *Staphylococcus warneri* | - | 1 | - | - | - | - |
| Total | 16 | 19 | 12 | 10 | 17 | 10 |

*Genus Micrococcus were identified using 16s rRNA sequencing

**Table S4: Species and numbers of bacteria isolated from females.**

| **Isolate species** | **Elderly females** | | | **Young females** | | |
| --- | --- | --- | --- | --- | --- | --- |
|  | **Hand** | **Foot** | **Scalp** | **Hand** | **Foot** | **Scalp** |
| *Acinetobacter baumannii* | - | - | 1 | - | - | - |
| *Aerococcus viridans* | - | - | - | - | 1 | - |
| *Burkholderia cepacia* | - | 1 | - | - | - | - |
| *Enterococcus faecalis* | - | - | 1 | - | - | - |
| *Enterococcus faecium* | - | - | - | - | 1 | - |
| *Klebsiella pneumoniae* | - | - | 1 | - | - | - |
| *Kocuria kristinae* | 1 | 2 | 1 | - | 1 | - |
| *Leuconostoc mesenteroides* | - | - | - | - | 2 | - |
| **Micrococcus spp.* | 3 | 4 | 2 | 3 | 9 | - |
| *Ochrobactrum anthropi* | - | - | - | - | - | 1 |
| *Pantoea spp.* | - | 2 | 1 | - | - | 2 |
| *Pseudomonas aeruginosa* | - | - | - | 1 | - | - |
| *Pseudomonas luteola* | - | 1 | - | - | - | - |
| *Raoultella ornithinolytica* | - | - | - | - | - | 1 |
| *Sphingomonas paucimobilis* | - | - | 1 | - | - | - |
| *Staphylococcus aureus* | 1 | - | 1 | - | - | - |
| *Staphylococcus capitis* | - | - | 1 | - | - | 1 |
| *Staphylococcus cohnii* | - | - | 1 | - | - | - |
| *Staphylococcus epidermidis* | 2 | - | 2 | 2 | - | 2 |
| *Staphylococcus haemolyticus* | 6 | 5 | - | - | - | - |
| *Staphylococcus hominis* | - | 2 | - | 1 | - | 1 |
| *Staphylococcus pasteuri* | - | 3 | - | - | - | - |
| *Staphylococcus warneri* | 3 | - | - | 1 | - | - |
| *Staphylococus xylosus* | 1 | - | - | - | - | - |
| Total | 17 | 20 | 13 | 8 | 14 | 8 |

*Genus Micrococcus were identified using 16s rRNA sequencing
